# *Beta vulgaris* ssp. *vulgaris* chromosome 8 shows significant association with geosmin concentration in table beet

**DOI:** 10.1093/g3journal/jkab344

**Published:** 2021-09-29

**Authors:** Solveig J Hanson, Julie C Dawson, Irwin L Goldman

**Affiliations:** 1 Centre for Sustainable Food Systems, The University of British Columbia, Vancouver, BC V6T 1Z4, Canada; 2 Department of Horticulture, University of Wisconsin—Madison, Madison, WI 53706, USA

**Keywords:** Table beet, *Beta vulgaris*, geosmin, association analysis, selective genotyping, QTL mapping

## Abstract

Geosmin, a degraded sesquiterpene molecule with earthy and musty odor, imbues table beet with its characteristic aroma. Geosmin is heritable and endogenously produced in table beet; its earthy aroma is sought by some consumers but deters others. Geosmin biosynthesis is catalyzed by a bifunctional geosmin synthase enzyme in diverse bacteria and fungi, but a mechanism for geosmin biosynthesis in plants has not been reported. This work employed association analysis and selective genotyping of a segregating F_2:3_ mapping population to seek QTL associated with geosmin concentration in table beet. GBS reads were aligned to sugar beet reference genome EL10.2, and association analysis revealed two QTL for geosmin concentration on *Beta vulgaris* ssp. *vulgaris* chromosome 8. QTL at EL10.2 positions 28,017,624 and 38,488,687 each show effect size 8.7 μ$g\cdot {\mathrm{kg}}^{-1}$ geosmin and explain 8.5% and 6.4% of total variation in geosmin concentration, respectively. Resolution was low due to large recombination bin size and imperfect alignment between the reference genome and mapping population, but population size and selection proportion were sufficient to detect moderate to large effect QTL. This study, the first molecular genetic mapping experiment in table beet, succeeded in finding QTL for geosmin concentration in table beet, and it provides the basis for fine mapping or candidate gene investigation of functional loci for this distinctive sensory trait.

## Introduction

Table beet (*B. vulgaris* ssp. *vulgaris*) is a minor biennial vegetable crop with significant regional and local economic impact. In Wisconsin, the United States’ largest table beet producing state, most beets are cultivated conventionally for processing markets (USDA NASS 2020) which demand standard red beets (Goldman and Navazio 2003). Beets are also an important crop for U.S. organic vegetable growers (Lyon *et al.* 2015; Brouwer and Colley 2016; Hultengren *et al.* 2016), who tend to prioritize flavor when selecting vegetable varieties (Dawson and Healy 2018). To facilitate breeding for both standard and novel table beet flavor characteristics, it is critical to understand the genetic basis of physicochemical components associated with flavor in table beet. Sweet and earthy flavor attributes are salient in table beet (Bach *et al.* 2014) and have been associated with sucrose (*e.g.*, Bach *et al.* 2014) and geosmin (Murray *et al.* 1975), respectively. Bitter flavor in table beet is associated with saponins (Mikołajczyk-Bator *et al.* 2016), flavonoids (Kujala *et al.* 2002), and phenols (Bavec *et al.* 2010). Raw beet can also produce astringent or pungent sensations (Bach *et al.* 2014), which may be associated with oxalate (Freidig and Goldman 2011), a compound known to produce abrasive sensations in other crops (Korth *et al.* 2006; Salinas *et al.* 2001).

The current work focuses on geosmin (*trans*-1,10-dimethyl-*trans*-9-decalol), the volatile 12-carbon terpenoid molecule that imbues moist soil with its distinctive aroma (Gerber 1967) and has been identified as the characteristic flavor of table beet (Tyler *et al.* 1979). Geosmin is present in a diverse group of terrestrial, aquatic, and plant-symbiotic microoroganisms (Churro *et al.* 2020), nonvascular plants like liverworts (Spiteller *et al.* 2002), and *B. vulgaris* ssp. *vulgaris* (Freidig and Goldman 2014), which includes crop types sugar beet, table beet, fodder beet, leaf beet, and Swiss chard (Letschert 1993). In table beet roots, geosmin is concentrated in and directly under the epidermis (Tyler *et al.* 1978; Lu *et al.* 2003a). Humans are exceedingly sensitive to geosmin, although perception thresholds for geosmin vary among individuals (Tyler *et al.* 1979), and preference for geosmin depends on consumption context. Geosmin is considered a contaminant in drinking water, wine, beer, and other foods (*e.g.*, Buttery *et al.* 1976; Frisvad *et al.* 1997; Darriet *et al.* 2000;), and anecdotal evidence exists both of nonpreference (Lu *et al.* 2003b) and preference (Goldman and Navazio 2008) for beet among consumers due to its earthy flavor.

While a mechanism for geosmin biosynthesis in *B. vulgaris* ssp. *vulgaris* has not been reported, geosmin biosynthesis has been confirmed in liverworts (Spiteller *et al.* 2002), fungi (Liato and Aïder 2017), actinobacteria, cyanobacteria, and proteobacteria (Churro *et al.* 2020). The biosynthesis of geosmin in *Streptomyces coelicolor* bacteria is a cyclization-fragmentation reaction catalyzed by a single bifunctional geosmin synthase enzyme in the presence of magnesium (Mg^2+^) ions. Sesquiterpene (C_15_) precursor molecule farnesyl diphosphate (FPP)—itself synthesized from ubiquitous C_5_ units (Singh and Sharma 2015)—is cyclized into an 85:15 mixture of germacradienol and germacrene D by the N-terminal domain of geosmin synthase. The enzyme’s C-terminal domain then catalyzes a fragmentation reaction which converts C_15_ germacradienol into C_12_ geosmin (Jiang *et al.* 2006) and C_3_ acetone (Harris *et al.* 2015).

The gene coding for geosmin synthase has been reported in *Streptomyces coelicolor* (Cane and Watt 2003) and model cyanobacterium *Nostoc punctiforme* (Giglio *et al.* 2008). The crystal structure of the *S. coelicolor* enzyme’s N-terminal domain has been determined, as has a homology model of the C-terminal domain (Harris *et al.* 2015). A complete geosmin synthesis gene cluster, composed of the geosmin synthase gene and two transcription regulator nucleotide binding genes, was discovered in the cyanobacteria *Microcoleus asticus*, and complete or partial geosmin synthase gene sequences were detected in actinobacteria, proteobacteria, and cyanobacteria occupying diverse terrestrial, aquatic, and symbiotic ecological niches (Churro *et al.* 2020). Dehydrogeosmin, a closely related sesquiterpene, is biosynthesized in the flowers of several *Cactaceae* species (Feng *et al.* 1993; Schlumpberger *et al.* 2004) using the same principal pathway as for geosmin biosynthesis in *Streptomyces* spp. (Spiteller *et al.* 2002). It is unknown whether the enzyme catalysts for these pathways arose independently or from a common ancestor.

A geosmin synthase gene has not been detected in *B. vulgaris*, but ample evidence exists that geosmin is endogenously produced in table beet and that its concentration is under primarily genetic control. Because geosmin is known to be synthesized by ubiquitous soil-dwelling microbes, it was assumed historically that geosmin was present in beet due to plant-microbe affiliation. However, table beet cultivars grown in autoclaved soil (Freidig and Goldman 2014) and aseptic tissue culture (Maher and Goldman 2018) were found to contain geosmin. Two experiments investigating genetic versus environmental control of geosmin in table beet cultivars—one comparing greenhouse and field treatments (Freidig and Goldman 2014) and one comparing differing field environments (Hanson and Goldman 2019)—found significant effects of genotype on geosmin concentration and mostly noncrossover genotype × environment interaction. Moreover, bidirectional recurrent selection for geosmin concentration in table beet was successful and produced realized heritability estimates of 0.70 and 0.23 for downward and upward selection, respectively (Maher and Goldman 2017). Hanson and Goldman (2019) showed broad sense heritability of 0.90 for geosmin concentration in a field-based genotype x environment experiment , adding to a substantial body of evidence establishing the primacy of genetic control over geosmin concentration in table beet. Geosmin concentration displays continuous variation consistent with multigenic control, and the variance of geosmin concentration is known to increase with concentration itself (Freidig and Goldman 2014; Maher and Goldman 2017; Hanson and Goldman 2019). However, genomic investigation is needed to elucidate the biosynthetic origin of geosmin in table beet.

Recent analyses of genetic structure within *B. vulgaris* ssp. *vulgaris* demonstrate that the genetic divergence of table beet from sugar, fodder, and leaf beet crop types is minor compared with their genetic similarity (Andrello *et al.* 2017; Galewski and Mcgrath 2020). No table beet genome has been published to date, but because sugar beet shows very close genetic relationship to table beet and is confirmed to contain geosmin (Tyler *et al.* 1978; Freidig and Goldman 2014), the genomic tools developed for sugar beet breeding can be leveraged for investigation of the genetic basis of geosmin concentration in table beet. Two sugar beet reference genomes have been published to date: RefBeet (Dohm *et al.* 2014) from short Illumina-derived DNA sequences and EL10 (Funk *et al.* 2018; McGrath *et al.* 2020) from PacBio long DNA reads, optical mapping, and Hi-C reads. EL10 versions EL10.1 and EL10.2 comprise nine main scaffolds, along with 31 and 9 additional small scaffolds, respectively. RefBeet and EL10.1 chromosomes are highly congruous and numbered according to conventional nomenclature (Butterfass 1964; Schondelmaier and Jung 1997). RefBeet chromosomes are oriented according to the cytogenetic map of Paesold *et al.* (2012), while chromosomal orientation is inverted for seven of nine EL10.1 chromosomes (McGrath *et al.* 2020). The EL10.2 genome assembly resolved inversions on EL10.1 chromosomes 7 and 9 and is the most contiguous sugar beet genome to date (McGrath *et al.* 2020).

The sugar beet genome is highly repetitive, characterized by concentrated regions of recombination, and subject to diverse forms of variation. Estimates of repetitive sequence in the sugar beet genome range from 42% (Dohm *et al.* 2014) to over 60% (Flavell 1974), with diverse repeat patterns among chromosomes (Paesold *et al.* 2012) and chromosomal recombination concentrated in certain genomic regions (*e.g.*, Barzen *et al.* 1992; McGrath *et al.* 2007; Dohm *et al.* 2014). Presence-absence variation—including indels, complex substitutions, and structural variation involving partial or entire genes—is a meaningful source of phenotypic variation and lineage divergence in sugar beet (Galewski and Eujayl 2021). Sugar beet genome size was estimated by flow cytometry to be 758 Mb (Arumuganathan and Earle 1991), but recent assemblies have fallen substantially short of that projection, ranging in size from 520 to 573 Mb (Galewski and Eujayl 2021). RefBeet and EL10.2 reference genomes have nearly identical size at 567 Mb (McGrath *et al.* 2020).

A preliminary search of the RefBeet genome (Dohm *et al.* 2014) for the *S. coelicolor* geosmin synthase protein sequence returned two hypothetical proteins with predicted terpenoid synthase function (Huang *et al.* 2017) located on *B. vulgaris* ssp. *vulgaris* chromosome 8 (Maher 2017). A probe using the *Solidago canadensis* germacrene-D synthase protein sequence yielded 56 potential genes, many of which had predicted sesquiterpene synthase function (Maher 2017). However, terpenoids are the largest and most diverse group of phytochemicals, making terpene synthase genes abundant and ubiquitous within plant genomes (Singh and Sharma 2015). In addition, geosmin synthase genes show evidence of rearrangements and deletions between bacterial phyla (Churro *et al.* 2020) which are much more closely related than bacteria and vascular plants, so sequence homology between geosmin synthase genes in *Streptomyces and B. vulgaris* cannot be assumed. While it is plausible that the *B. vulgaris* ssp. *vulgaris* genome harbors a geosmin synthase gene, additional evidence is required to establish its location and functionality.

Genetic mapping has been used for decades to detect association between genetic markers and genomic loci that influence phenotypes. Linkage mapping was first used in sugar beet to detect qualitative traits like hypocotyl color (Barzen *et al.* 1992; Pillen *et al.* 1992), fertility restoration (Pillen *et al.*^1992^, ^1993^), monogermy and Rhizomania resistance (Barzen *et al.* 1992), and bolting (El-Mezawy *et al.* 2002). Efforts to detect quantitative traits like sugar yield and quality met with limited success (Weber *et al.* 1999; Weber *et al.* 2000) due to significant environmental variation, low marker density, and the lack of consistent genetic polymorphism across populations. A few sugar beet linkage mapping projects succeeded in identifying QTL potentially useful for marker-assisted selection (*e.g.*, Rajabi and Borchardt 2015), but limitations remained to this approach, particularly low resolution due to large linkage blocks in biparental segregating populations, and limitation to two alleles per locus (Flint-Garcia *et al.* 2003). Association analysis is often used with diverse panels which have rapid decay of linkage disequilibrium. Such an analysis using elite sugar beet breeding lines and wild beet accessions showed that linkage disequilibrium varied by both population history and by chromosome (Adetunji *et al.* 2014). Association analysis requires control of population structure to identify true marker-trait associations, and in sugar beet, population structure has been sufficiently controlled by including the kinship matrix as a random effect in the marker-trait association model (Würschum *et al.* 2011; Mangin *et al.* 2015).

Selective genotyping, the practice of genotyping only individuals with extreme phenotypes, can be used for QTL detection (Navabi *et al.* 2009). It was first used in bulk segregant analysis (Stuber *et al.* 1980; Zou *et al.* 2016), and Lander and Botstein (1989) used this technique in linkage mapping experiments to control costs while retaining a high percentage of linkage information. Bidirectional selective genotyping—selecting equal proportions from each tail of the phenotypic distribution—maximizes both power to detect QTL (Navabi *et al.* 2009) and efficiency compared with complete population genotyping (Rabier 2014). For a given proportion of individuals selected for genotyping, power increases with QTL effect size, total population size, and proximity of QTL to markers (Navabi *et al.* 2009). Optimum selection proportion may be calculated as a function of the relative costs of genotyping and phenotyping for a given experiment (Darvasi and Soller 1992; Gallais *et al.* 2007) or using evidence from simulation studies (Rabier 2014; Sun *et al.* 2010).

The current work seeks to locate genomic loci associated with table beet geosmin concentration to connect the physical beet genome with existing statistical and observational evidence that this trait is under primarily genetic control. Identification of QTL for geosmin concentration in table beet could also yield insight into the mechanism of geosmin biosynthesis in beet and facilitate development of molecular markers for table beet flavor breeding. This is the first molecular marker-based genetic mapping effort in table beet to our knowledge, and it is unique among *B. vulgaris* ssp. *vulgaris* mapping projects for its focus on a sensory trait rather than one important for commodity food manufacturing.

## Methods

### Germplasm

A population of F_3_ individuals segregating for geosmin concentration was developed from a biparental cross of two individuals from open pollinated table beet cultivars. Seed parent cultivar Touchstone Gold’ and pollen parent cultivar ‘Chioggia are known to have relatively low and high mean geosmin concentration, respectively (Freidig and Goldman 2014). A single F_1_ plant was self-pollinated to produce a segregating F_2_ family, and 194 F_2_ plants were self-pollinated to create F_2:3_ families. Populations of parental cultivars Touchstone Gold’ and Chioggia’ were maintained through mass pollination and used as check cultivars.

### Field and greenhouse methods

Seed for the F_2:3_ mapping population was produced in three consecutive annual cycles, each composed of summer field-based root production and winter greenhouse seed production. Parental, F_1_, and F_2_ roots were harvested in late August 2014, 2015, and 2016, respectively. Parents were co-isolated to achieve the initial biparental cross in February 2015, and single plants were bagged to achieve F_1_ and F_2_ self-pollinations in February 2016 and 2017, respectively. Seed was hand harvested from each plant; of 194 F_2:3_ families self-pollinated in 2017, 90 produced sufficient seed for inclusion in the mapping population. The mapping population of 90 F_2:3_ families was grown in two randomized replications in both 2017 and 2019. Four plots each of parental cultivars Touchstone Gold’ and Chioggia’ were placed randomly in each replication, for 98 total entries per replicate. Plots measuring 1.2 m long and spaced 46 cm apart were hand-seeded in 2017 and transplanted in 2019.

Three individuals free from surface damage, with root diameter 4 to 6.5 cm, and with tops intact were harvested from each plot 11.5 weeks after direct seeding in 2017 and 8.5 weeks after transplanting in 2019. Leaf and root tissue were sampled from 2 individuals per F_2:3_ family plot and one root per check plot within 2 days of harvest. For each leaf sample, five 1-cm disks were punched from a single newly emerged mature leaf and frozen at −80°C in either fresh or lyophilized form. Roots were dry-brushed to remove soil, and a core borer with 1 cm internal diameter was used to extract 6 to 10 cylindrical cores, each with 1 to 3cm length and an epidermal disk of approximately 1 cm diameter. Cores were frozen immediately at −80°C and transferred to a −20°C freezer before laboratory analysis. 84 of 90 F_2:3_ families yielded sufficient roots for sampling.

### GC-MS phenotyping

Geosmin concentration was calculated via HSPME and GC–MS using the method described by Hanson and Goldman (2019) and Maher and Goldman (2017, 2018), adapted from Lu *et al.* (2003b). Briefly, beet homogenate was prepared for each root tissue sample using six root cores with complete epidermal disks. Cores were trimmed to achieve a 30 g sample mass, combined with Milli-Q water (Millipore, Bedford, MA, USA) at a 1:1 ratio, and blended for 2 min in an industrial blender. Homogenate was held at −20°C until immediately before GC-MS analysis, when it was thawed slightly in a 32°C water bath. Partially frozen homogenate was combined with NaCl in a screw-top glass vial at a 5 g : 1 g ratio, and the vial was sealed immediately with a polytetrafluoroethylene (PTFE)-silicone septum-lined cap. A water blank and three geosmin standards of 5, 10, and 21.6 $\mu g\cdot {\mathrm{kg}}^{-1}$ ± (–)geosmin (Sigma-Aldrich, St. Louis, MO) in 5 g Milli-Q water were included with each GC–MS cycle for calibration curve generation. Internal standard –(–)menthone (Sigma-Aldrich) was injected at the rate of 2.82 $\mu g\cdot {\mathrm{kg}}^{-1}$ root tissue in all table beet samples and geosmin standards. Relative recovery was calculated by comparing geosmin concentration in unmodified table beet homogenate with that in identical homogenate spiked with 5, 10, 15, and 21.6 $\mu g\cdot {\mathrm{kg}}^{-1}$ geosmin, according to the method of Lu *et al.* (2003b). Relative recovery rates for 2017 and 2019 were 40.37% and 46.76%, respectively.

$$\mathrm{Relative}\;\mathrm{Recovery}=\;\frac{{\mu g\;\mathrm{Geosmin}\;}_{\mathrm{Total}}-\;{\mu g\;\mathrm{Geosmin}\;}_{\mathrm{Unmodified}}\;}{{\mu g\;\mathrm{Geosmin}\;}_{\mathrm{Spiked}}}\;\times 100\%$$

### Phenotypic data analysis

Phenotypic data for the full mapping population, selectively genotyped population, and check plots were analyzed using R software (R Core Team 2021). Mixed model ANOVA and Tukey-corrected pairwise comparisons with experiment-wide error rate α=0.05 were conducted with R packages “lme4” (Bates *et al.* 2015), “lmerTest” (Kuznetsova *et al.* 2017), and “emmeans” (Lenth 2020). The statistical model used was:

$${\text{Geo}}_{\text{ijkl}}={\;\text{Year}}_{i}+{\;\text{Family}}_{j}+{\left(\text{Year}*\text{Family}\right)}_{\text{ij}}+{\;\text{Block}}_{k}{\left(\text{Year}\right)}_{i}+{\left(\text{Block}*\text{Year}*\text{Family}\right)}_{\text{ijk}}+\varepsilon_{\text{ijkl}}$$

where Geo_ijkl_ is individual root geosmin concentration; Year_i_ is the fixed effect of year i, i = {2017, 2019}; Family_j_ is the fixed effect of family j, j = {1…78}; Block_k_ is the fixed effect of block k, which was nested within year, *k* = {1,2}. The random Block*Year*Family interaction term expressed plot-to-plot variation and was used as the denominator for significance tests of fixed effects.

Welch *T*-tests were used for comparison of mean geosmin concentration between years, between check plots and F_2:3_ populations, and between high and low geosmin tails of the selected population. Log-transformed data conformed better to ANOVA assumptions and were used for all statistical tests. Data were visualized using R package “ggplot2” (Wickham 2016). Of the 84 phenotyped families, 6 yielded fewer than two roots in 2017 and were excluded from the full mapping population.

### Selective genotyping

Selective genotyping was performed on a subpopulation of F_3_ individuals representing 16% of the full mapping population, divided equally between tails of the geosmin concentration distribution. Because 2017 and 2019 populations were of different sizes, and because a significant Year effect was observed with respect to geosmin concentration, 2017 and 2019 populations were considered separately for the purpose of individual selection. F_2:3_ families were ranked by mean geosmin concentration within year, and a subset of families was identified that showed geosmin concentration in the extreme quartile of the distribution in both years. Next, F_3_ individuals were ranked by geosmin concentration within the year of production. Individuals were considered for selection beginning with the most extreme individual and working toward the mean; individuals were selected only if they belonged to one of the families with extreme mean geosmin concentration in both 2017 and 2019.

The original selected population (*n* = 92) was composed of 40 individuals from 2017 and 52 individuals from 2019, divided equally between high and low geosmin tails within year. Four genotyped individuals were excluded from further analysis because they belonged to families with too few 2017 roots for valid ANOVA, and one genotyped individual yielded insufficient DNA for analysis. The analyzed population (*n* = 87) consisted of a low geosmin tail (*n* = 43) of 20 and 23 individuals from 2017 and 2019, respectively, and a high geosmin tail (*n* = 44) of 19 and 25 individuals from 2017 and 2019, respectively.

A single 1-cm disk of leaf tissue was submitted to the University of Wisconsin Biotechnology Center (Madison, WI) for DNA extraction and genotyping by sequencing (Elshire *et al.* 2011). DNA libraries were prepared by digestion with *NsiI-BfaI* and ligation of GBS barcodes and adapters. Paired-end reads (2 × 150 bp) were generated on one shared lane of an Illumina NovaSeq 6000 sequencer.

### Genomic data analysis

Genomic data quality control, sequence alignment, and SNP calling were performed by the University of Wisconsin Bioinformatics Resource Center (Madison, WI, USA). Skewer software (Jiang *et al.* 2014) was used to remove adapters, primers, and low-quality bases, to trim reads until achievement of Phred quality 20, and to discard excessively short reads. Demultiplexed 64 bp forward reads were aligned to *B. vulgaris* ssp. *vulgaris* reference genome EL10.2 (McGrath *et al.* 2020) using the Tassel v2 GBS Pipeline (Glaubitz *et al.* 2014) and alignment software Bowtie 2 (Langmead *et al.* 2012). Variants were called using the Tassel v2 Discovery and Production SNP Caller system. 185,457 unfiltered SNP variants were detected.

VCF files were filtered using VCFtools (Danecek *et al.* 2011) to eliminate indel mutations and retain only biallelic SNP’s with minor allele frequency >0.10, read depth ≥5, and no missing individuals. Preliminary analyses that included EL10.2 small scaffolds 10–18 revealed no SNP’s close to the significance threshold, so SNP’s on these contigs were omitted for clarity, resulting in a set of 36,928 markers. SNP markers were filtered in R by genotype frequency to eliminate sites with <5% minor homozygotes and >50% heterozygotes. Marker data were analyzed for linkage disequilibrium within chromosome (Rogers and Huff 2009), and a single marker was selected to represent groups of markers with LD >0.99. Bins containing markers from multiple chromosomes were examined individually, and ambiguously placed markers were eliminated, resulting in a final set of 8651 markers. EL10.2 main scaffolds were renumbered to bring them into accordance with Butterfass chromosome naming convention. Haplotypes were not phased due to unavailability of parental tissue for sequencing, so the reference and alternate alleles were designated 0 and 1, respectively, based on comparison with reference genome EL10.2. Deviations from the reference genome are useful for detecting genomic loci associated with geosmin but not for assigning such deviations to parental genotypes.

While biparental populations are not expected to exhibit population structure, selective genotyping could introduce it. Thus, population structure was analyzed and visualized using principal components analysis (PCA) with base R software and package “ggplot,” respectively. Single marker analysis was performed using R package “GWASpoly” v.2.07 (Rosyara *et al.* 2016) with settings for diploid organisms, additive gene action, and significance threshold set using 1000 permutations (Churchill and Doerge 1994) to control the family-wise error rate at α=0.05. A separate relationship matrix (K) was calculated for each chromosome using the “leave-one-chromosome-out” method (Yang *et al.* 2014) to account for the random effect of genotype (Würschum *et al.* 2011; Mangin *et al.* 2015). The most significant marker in each 1 Mb window was reported, and a QTL model was fit to this marker subset in “GWASpoly” using backward regression. F_3_ individual geosmin concentration was used as the phenotypic variable, but because F_3_ individuals are not replicable, the F_2:3_ family mean in both years was taken into account during selection of individuals for genotyping. Because the permutation-derived threshold is independent of phenotypic data distribution, untransformed geosmin concentration data could be used for analysis, simplifying interpretation of results.

## Results

### Mapping population

**Figure 1:**
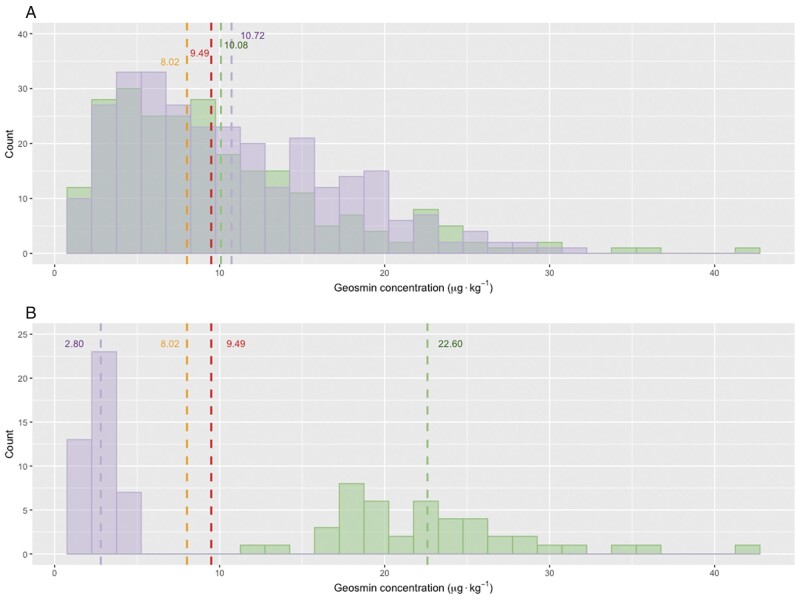
Distribution of geosmin concentration in (A) a mapping population of F_3_ individual table beets grown in 2017 (green) and 2019 (purple) and (B) high (green) and low (purple) F_3_ individuals selected for genotyping. Mean geosmin concentration for low and high geosmin check plots are shown in gold and red font, respectively.

The distribution of untransformed geosmin concentration in the full mapping population was right-skewed for both individual roots (Figure 1) and F_2:3_ family means (data not shown). Full populations from 2017 and 2019 did not differ significantly in mean geosmin concentration when compared using individual roots (Welch *T* = −1.45 on 517.6 DF, *P* ≤ 0.15) or family means (Welch *T* = −0.99 on 152.9 DF, *P* ≤ 0.32). Mixed model ANOVA performed on individual geosmin concentration values showed a significant Year effect and extremely significant Family effect (Table 1). Pairwise comparisons among F_2:3_ families were used to confirm the extreme-geosmin nature of families prioritized for selective genotyping (Supplementary Table S1). Each of the 78 F_2:3_ families in the mapping population was represented by between 4 and 8 individuals, with mean 6.95 individuals per family.

**Table 1:** ANOVA for log transformed geosmin concentration of a segregating table beet population composed of 542 individual roots from 78 F_2:3_ table beet families, grown in 2017 and 2019

Source of Variation	SS	MS	NumDF	DenDF	*F* value	Pr(>F)
Year	1.25	1.25	1	109.8	6.29	0.01*
Family	120.39	1.56	77	104.4	7.88	<2e-16***
Year: Block	0.51	0.26	2	110.0	1.29	0.28
Year: Family	19.42	0.25	77	104.4	1.27	0.13

*, *** Significant at *P* < 0.05 and 0.001, respectively

Plots of high geosmin check Chioggia’ were numerically but not significantly higher in mean geosmin concentration than those of low geosmin check ‘Touchstone Gold’ at 9.49 and 8.02 $\mu g\cdot {\mathrm{kg}}^{-1}$, respectively, averaged over years. Mean geosmin concentration of the full population of F_3_ individuals, averaged over years, was not significantly different from that of high geosmin check ‘Chioggia’ (Welch *T* = 0.95 on 12.8 DF, *P* ≤ 0.36) nor low geosmin check Touchstone Gold’ (Welch *T* = −0.29 on 17.3 DF, *P* ≤ 0.78). It should be noted that while check populations are of the same cultivar as parental individuals, actual geosmin concentration of parental individuals is unknown.

High and low geosmin tails (*n* = 44 and *n* = 43, respectively) were selected to include F_3_ individuals with extreme-quartile geosmin concentration in the year of their production, as well as extreme-quartile F_2:3_ family mean geosmin concentration in both 2017 and 2019 (Supplementary Table S2). All selected individuals were from F_2:3_ families with two-year mean geosmin concentration in the most extreme pairwise comparison group (Supplementary Table S1). 24 F_2:3_ families were represented in the selected population, with a range of 1 to 6 individuals per family (Supplementary Table S3). High and low geosmin tails showed extremely significant differences in mean geosmin concentration, at 22.60 and 2.80 $\mu g\cdot {\mathrm{kg}}^{-1}$ geosmin concentration, respectively (Welch *T* = 31.95 on 75.3 DF, *P* ≤ 2.2 × 10^−16^) (Figure 1). The high geosmin tail of the selected population was higher in geosmin concentration than high geosmin check ‘Chioggia’ (Welch *T* = 7.99 on 18.7 DF, *P* ≤ 1.93 × 10^−07^), and likewise, the low geosmin tail of the selected population was significantly lower in geosmin concentration than low geosmin check Touchstone Gold’ (Welch *T* = −8.89 on 17.3 DF, *P* ≤ 7.18 × 10^−08^).

### Population structure

PCA was performed using genotypic data from the selected individuals at the 8,651 biallelic SNP markers used for association analysis. The percent of total genotypic variation explained by each dimension declined smoothly from a maximum of 18.4% variation, with no clear inflection point. PCA plots showed no obvious clustering by low or high geosmin tail. Some clustering by F_2:3_ family was evident, but family clusters were highly overlapping and distributed over many dimensions (Supplementary Figure S1). Moreover, the full mapping population appeared as a cloud of points along plots of all combinations of dimensions 1–4 (Supplementary Figure S2).

Families with particularly extreme geosmin concentration were represented by multiple individuals in the genotyped population, while others were included only once, so population structure could have been higher than in a population composed of one individual per F_2:3_ family. To investigate this possibility, a balanced subpopulation was created using only one individual from each of the 24 F_2:3_ families in the selected population. The first dimension explained 24.8% of genotypic variation—slightly more than in the full genotyped population—and percent variation declined more steeply between first and second dimensions, demonstrating marginally more population structure than the full genotyped population, rather than less (Supplementary Figure S3). Thus, population structure in the genotyped population was quite minimal and required no further correction than that provided by the relationship matrix (K).

### Association analysis

**Figure 2:**
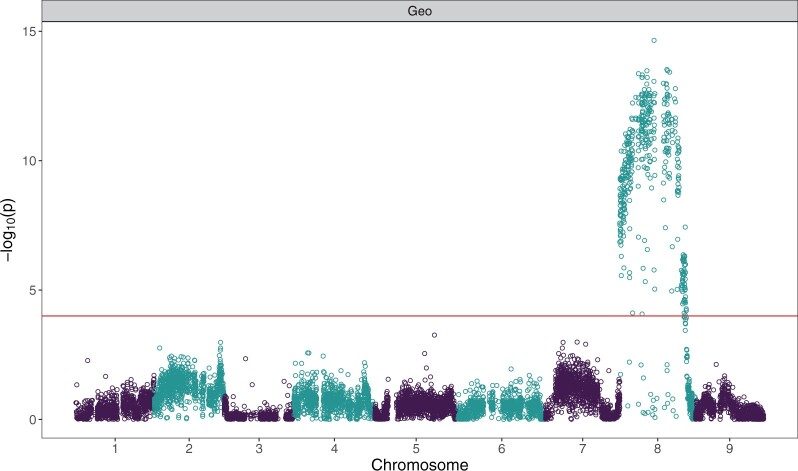
Manhattan plot for association analysis performed with respect to geosmin concentration on 8,651 SNP markers from a table beet population selected for extreme geosmin concentration (*n* = 87 F_3_ individuals) using −log_10_(*p*) = 4.0 significance threshold derived from 1000 permutations (α  =  0.05).

Association analysis revealed a large portion of *B. vulgaris* ssp. *vulgaris* chromosome 8 to be significantly associated with geosmin concentration. Markers exceeding a −log_10_(*p*) = 4.0 significance threshold derived from 1000 permutations (α  =  0.05) were detected in 22 one-Mb windows, spanning all but the last 10 Mb of the 62 Mb chromosome (Figure 2). For windows with significant markers, the most significant marker was reported, along with its effect size (Table 2). QQ plots confirm association of many markers on chromosome 8 with geosmin concentration but show no evidence of association on other chromosomes (Supplementary Figure S4). Markers were tested for inclusion in a QTL model using backward regression, and two QTL—at EL10.2 positions 28,017,624 and 38,488,687—were found to explain 8.5% and 6.4% of total variation in geosmin concentration, respectively. Taken together, all significant markers explain 19.1% of variation in geosmin concentration.

**Table 2:** **Markers with maximum significance for each 1 Mb window in which any significant markers were detected** Association analysis was performed on a table beet population selected for extreme geosmin concentration (*n* = 87 F_3_ individuals) using 8651 SNP markers and −log_10_(*p*) = 4.0 significance threshold derived from 1000 permutations (α  =  0.05). Markers were tested for inclusion in a QTL model using backward regression, and percent variation in geosmin concentration explained (*R*^2^) and *P* values are shown.

Marker	Chr	EL10.2 Position	Score	Effect	*R* ^2^ (%)	*P*
8_1205464	8	1205464	10.37	6.8	0.0	0.93
8_4759765	8	4759765	11.03	7.3	0.0	1
8_5944761	8	5944761	10.93	7.3	0.0	1
8_8471698	8	8471698	11.21	7.3	0.4	0.57
8_10406017	8	10406017	12.23	8.0	0.0	1
8_13243064	8	13243064	12.44	8.2	0.0	1
8_15304025	8	15304025	13.36	8.2	0.0	1
8_18397052	8	18397052	13.31	8.7	0.1	0.78
8_20033066	8	20033066	12.75	8.4	0.0	1
8_22165325	8	22165325	13.48	8.8	0.0	1
8_23775128	8	23775128	12.75	8.4	0.0	1
8_25275567	8	25275567	12.59	8.2	2.3	0.15
8_28017624	8	28017624	14.64	8.7	8.5	5.45e-03**
8_35673306	8	35673306	12.99	8.4	0.0	0.91
8_38488687	8	38488687	13.52	8.7	6.4	1.64e-02*
8_40730112	8	40730112	13.42	8.5	0.0	1.00
8_42812663	8	42812663	11.6	7.6	0.0	0.88
8_45449861	8	45449861	12.78	8.4	0.5	0.49
8_47543727	8	47543727	10.87	7.0	0.0	0.97
8_50251664	8	50251664	5.76	−4.8	0.3	0.61
8_51746401	8	51746401	6.36	−5.6	0.5	0.52
8_53413075	8	53413075	7.43	−5.5	0.1	0.81

*, ** Significant at *P* < 0.05 and 0.01, respectively

For the 22 markers representing one-Mb windows in which significant markers were detected, mean effect size was 7.7 $\mu g\cdot {\mathrm{kg}}^{-1}$ geosmin concentration, ranging in absolute value from 4.8 to 8.7 $\mu g\cdot {\mathrm{kg}}^{-1}$ geosmin concentration (Table 2). Effect size was maximized at the two QTL identified for model inclusion, along with a third at EL10.2 position 18,397,052 that added only marginal explanatory value.

The validity of this analysis was supported by separate association analyses on the F_3_ individuals comprising the low (*n* = 43) and high (*n* = 44) geosmin tails of the selected population using the same set of 8651 markers. These analyses function as negative controls, as no significant marker-trait association should be detected when individuals from only one tail of the geosmin concentration distribution are analyzed. Population structure of the entire genotyped population and each single-tail subpopulation was similar, confirming that any population structure shown in the entire genotyped population was unrelated to geosmin concentration. (Supplementary Figure S3). For both high and low geosmin tail populations, no markers exceeded the α  =  0.05 permutation-derived significance threshold (Supplementary Figure S5). Individual-chromosome QQ plots associated with these analyses show mostly depressed Observed −log_10_(*p*) values at the upper end of the Expected −log_10_(*p*) value distribution, rather than amplified values that would indicate significant marker-trait association as in the main analysis (Supplementary Figure S6). Thus, support accrues for the result of the main association analysis: that two QTL on chromosome 8 are truly associated with geosmin concentration in table beet.

## Discussion

This analysis, the first molecular marker-based genetic mapping in table beet, offers strong evidence of QTL for geosmin concentration on *B. vulgaris* ssp. *vulgaris* chromosome 8. By establishing a genomic location associated with geosmin—the compound that imbues table beet with its distinctive earthy aroma—this study links evidence of endogenous production of geosmin in table beet (Maher and Goldman 2018) with evidence of genetic control of this trait (Freidig and Goldman 2014; Maher and Goldman 2017; Hanson and Goldman 2019). As such, it provides a starting point for fine mapping or direct gene identification strategies that could identify a functional gene responsible for geosmin biosynthesis in table beet.

While this analysis detected significant markers in 22 1 Mb windows spanning much of chromosome 8, only two QTL explained most of the observed variation in geosmin concentration. That is, significant markers excluded from the QTL model may be physically linked to the major QTL due to the large recombination blocks inherent in this F_2:3_ mapping population. The chromosomal positions of the major QTL should be considered in context of the repetitive nature of the *B. vulgaris* ssp. *vulgaris* genome and the imperfect alignment of the reference genome with reads from the mapping population. The beet genome shows substantial presence-absence variation (Galewski and Eujayl 2021) and is highly repetitive (Flavell 1974; Dohm *et al.* 2014), with the nature and size of inserted, deleted, and repeated regions varying among individuals and genotypes. Moreover, table beet is the most genetically distinct crop type within *B. vulgaris* ssp. *vulgaris*, harboring more lineage-specific variation than other *B. vulgaris* ssp. *vulgaris* crop types (Galewski and McGrath 2020). While the EL10.2 genome was constructed from long reads and used several technologies to increase the probability of correct sequencing in repetitive regions, it is entirely plausible for repetitive regions in this table beet mapping population to be translocated, inverted, multiplied, or deleted with respect to the EL10.2 sugar beet reference genome. Due to imperfect alignment, large recombination bin size, and decreased effectiveness of alignment algorithms in highly repetitive regions (Treangen and Salzberg 2011), these QTL positions should be interpreted cautiously.

While this mapping population does not lend itself to precise QTL positioning, it is clear that QTL for geosmin concentration are located on *B. vulgaris* ssp. *vulgaris* chromosome 8. Interestingly, chromosome 8 was also found to harbor two adjacent hypothetical proteins with predicted terpenoid synthase function (BVRB8g184050 and BVRB8g184060) showing more than 20% identity with the *S. coelicolor* geosmin synthase protein sequence. In the EL10.1 genome, the nucleotide sequence of BVRB8g184050 encompasses two genes (*EL10Ac8g20305* and *EL10Ac8g20306*), both of which encode alpha-farnesene synthase 1 (AFS1) proteins (Phytozome 2021). The sequence of BVRB8g184060 co-locates with much of *EL10Ac8g20304* and is adjacent to *EL10Ac8g20302*, both of which code for S-linalool synthases (Phytozome 2021). All four EL10.1 genes are functionally annotated as terpenoid cyclases, and three of four genes (*EL10Ac8g20302*, *EL10Ac8g20304*, and *EL10Ac8g20305*) include sequences annotated as terpene synthase metal-binding domains. Biosynthesis of geosmin in species of actinobacteria, cyanobacteria, and fungi is driven by a bifunctional Mg^2+^-binding sesquiterpene synthase enzyme (Jiang *et al.* 2006; Giglio *et al.* 2008; Churro *et al.* 2020), so the functional homology of these putative *B. vulgaris* ssp. *vulgaris* terpenoid synthases is worth noting. However, terpene synthase enzymes are ubiquitous within plant genomes (Singh and Sharma 2015), and it cannot be assumed that these genes code for functional geosmin synthases.

Taken together, these putative terpenoid synthase genes make up a region approximately 123 kb long, located about 5 Mb from the end of EL10.1 chromosome 8, consistent with the phenomenon of clustered plant biosynthetic genes (Huang *et al.* 2017). Both QTL for geosmin concentration are located near the center of EL10.2 chromosome 8, at approximate positions of 28 and 38 Mb. Estimation of the distance between QTL and this cluster of terpenoid synthase genes is complicated by the fact the orientation of EL10.2 chromosomes is not established—either with respect to EL10.1 or the cytogenetic map—and the size of chromosome 8 differs between EL10.1 and EL10.2 (McGrath *et al.* 2020). Thus, while QTL for geosmin concentration do not obviously co-locate with terpenoid synthase genes showing functional similarity to *S. coelicolor* geosmin synthase, it is notable that both elements are located on chromosome 8.

While this study detected two major QTL for geosmin concentration on *B. vulgaris* ssp. *vulgaris* chromosome 8, the distribution and variance of geosmin concentration in the full mapping population was consistent with that of a quantitative trait. Geosmin concentration exhibited continuous distribution ranging from 0.77 to 41.60 $\mu g\cdot {\mathrm{kg}}^{-1}$geosmin, along with right skew, demonstrating the established phenomenon that variance of geosmin concentration tends to increase with concentration itself (Freidig and Goldman 2014; Maher and Goldman 2017; Hanson and Goldman 2019). The full population was created by crossing two parental individuals with moderate and unknown geosmin concentration, and the resulting transgressive segregation—in which a segregating population shows more extreme phenotypes than those of either parent—could owe to a combination of additive, dominance, and epistatic effects. If QTL for geosmin concentration does code for geosmin synthase enzymes, the observed continuous variation in geosmin concentration could owe to the presence of multiple loci harboring geosmin synthase genes, per an additive effects model (Lynch and Walsh 1998). In addition, multiple geosmin synthase alleles could exist, the functionality of which could differ if mutations to functional domains altered their affinity for Mg^2+^ cofactors (Harris *et al.* 2015), sesquiterpene substrates, or both. If a *B. vulgaris* ssp. *vulgaris* geosmin synthase gene complex included transcription factor binding genes like that of *M. asticus* (Churro *et al.* 2020), mutations in these genes could alter geosmin synthase activity. Due to the ubiquity of C_5_ and C_15_ compounds within plant tissues (Singh and Sharma 2015), geosmin biosynthesis seems unlikely to be affected by substrate availability.

It is also possible that some QTL for geosmin concentration could have gone undetected in this analysis. In selective genotyping experiments, power to detect QTL is influenced by the overall population size, the proportion of individuals selected for genotyping, and the effect size of QTL associated with the trait (Navabi *et al.* 2009; Sun *et al.* 2010). In the present experiment, a 16% selection proportion from a population of 542 individuals allowed detection of QTL explaining 8.5% and 6.4% of variation in geosmin concentration. These effect sizes are consistent with those from simulation studies using similar selected proportions and population sizes (Navabi *et al.* 2009; Sun *et al.* 2010). However, larger overall population size and possibly larger selected proportion would have been needed to detect smaller-effect QTL. This analysis did detect some small-effect QTL—such as the marker at EL10.2 position 25,275,567 that explained 2.3% of variation in geosmin concentration—but it may have lacked the power for such QTL to be included in a regression model. In addition, since this population’s parents were from open pollinated cultivars rather than inbred lines, and since ‘Touchstone Gold’ and ‘Chioggia’ are not extremely different in geosmin concentration, it is likely that this mapping population did not segregate at all loci related to geosmin concentration in table beet.

This study detected two QTL with estimated effect size 8.7 $\mu g\cdot {\mathrm{kg}}^{-1}$ geosmin concentration, but numerical estimates of effect sizes should be viewed as approximate. It is well known that the statistical methods used for association analysis can cause overestimation of QTL effects, especially in relatively small population sizes like that used in this experiment (Beavis 1998; Xu 2003). In addition, Darvasi and Soller (1992) caution that QTL effects are overestimated when a selective genotyping approach is used. Lee (2005) shows that unidirectional selective genotyping for marker-based analysis can reduce accuracy of QTL detection and produce downward bias in QTL effects estimation, especially when selection is made within full-sib families. While Manichaikul *et al.* (2007) found that QTL effect sizes are not inflated under bidirectional selection, Navabi *et al.* (2009) demonstrate slight under- and overestimation of QTL effect size using unidirectional and bidirectional selection. Given the inconsistency of simulation results, the fact that this experiment selected individuals both among and within F_2:3_ families, and the fact that individual phenotypes were not replicated, estimated QTL effect sizes should be interpreted with caution. However, to the degree that effect sizes of 8.7 $\mu g\cdot {\mathrm{kg}}^{-1}$ geosmin are accurate, they would be commensurate with the approximate annual change in population mean geosmin concentration under recurrent selection for that trait; preliminary taste tests are inconclusive with respect to whether this magnitude of difference in geosmin concentration is perceptible to consumers (Maher and Goldman 2017). Because this experiment used unphased sequence data, it cannot be known whether alternate alleles originated from the ‘Touchstone Gold’ or Chioggia’ parent.

To validate the association of chromosome 8 QTL with geosmin concentration in table beet, and to more precisely locate QTL, fine mapping could be carried out with either a linkage or association approach. A linkage approach could increase power to detect QTL by creating a segregating F_2_ population from a cross of two parents with widely divergent geosmin concentration, which are now available due to the recurrent selection work of Maher and Goldman (2017) and participatory cultivar development work of Hanson (2020). Parental roots could be genotyped to facilitate haplotype phasing, and F_2_ individuals could be genotyped and paired with mean F_2:3_ family phenotypes to reduce phenotypic error variance. Such a population would facilitate composite interval mapping, which should yield a much smaller QTL region than did this single marker analysis. An association approach incorporating accessions with diverse recombination histories and widely variable geosmin concentration could also be used for fine mapping of loci controlling geosmin concentration in table beet.

Direct gene identification could also be pursued via either allele frequency analysis or a candidate gene strategy. Allele frequency analysis using bulked sequence data is particularly effective in *B. vulgaris* ssp. *vulgaris* due to its genetic heterogeneity and the fact that phenotypes are both changed and measured on a population mean basis (Ries *et al.* 2016; Tränkner *et al.* 2016; Galewski and Mcgrath 2020). This trait-based selective genotyping strategy could be applied to any population yielding two pools with extreme geosmin concentration, including those created for linkage mapping or GWAS. Alternatively, candidate geosmin synthase genes—perhaps including those identified by Maher (2017) and additional chromosome 8 sequences encoding predicted terpene synthases—could be validated against sequence data from extreme-geosmin individuals. Finally, RNA-seq could be used to identify candidate genes with differential expression levels between high and low geosmin genotypes, or between epidermal and parenchymal tissues of the same root.

This study was the first molecular marker-based genetic mapping experiment in table beet, and it succeeded in detecting two QTL for geosmin concentration in table beet. This finding adds to the body of evidence documenting the genetic basis of geosmin concentration in table beet, and it provides a starting point for more thorough investigation of chromosome 8 with fine mapping or direct gene identification strategies. If successful, such strategies might locate one or more genes encoding *B. vulgaris* ssp. *vulgaris* geosmin synthase, allowing development of molecular markers for this trait. While more research is warranted into consumer preference for geosmin concentration—and more broadly, into consumer flavor perception and hedonic liking for table beet—manipulation of geosmin concentration in table beet offers one way to adjust the physicochemical composition of table beet and therefore, plausibly, its perceived flavor profile, consumer acceptance, and market potential.

## Data availability

Code and raw data are available via figshare at https://doi.org/10.25387/g3.16649746 (Accessed: 2021 October 1). Supplementary File S1 contains R code used to analyze phenotypic data found in Supplementary Files S2 and S3. Supplementary File S4 contains R code used for association analysis and PCA. Association analysis uses phenotypic data found in Supplementary File S5, and PCA uses categorical data in Supplementary File S6. Both analyses use the filtered marker data set in Supplementary File S7. Unfiltered genomic data in Variant Call Format are found in Supplementary File S8. Supplementary Files S7 and S8 include original EL10.2 scaffold names that were later renamed according to Butterfass nomenclature. Aligned population sequence data is available in the NCBI SRA database at https://www.ncbi.nlm.nih.gov/sra/?term=PRJNA764531 (Accessed: 2021 October 1).
